# Morpho-Orthographic Complexity in Affix Spelling in Hebrew: A Novel Psycholinguistic Outlook Across the School Years

**DOI:** 10.3389/fpsyg.2020.00868

**Published:** 2020-05-26

**Authors:** Rachel Schiff, Shlomit Rosenstock, Dorit Ravid

**Affiliations:** ^1^School of Education, Faculty of Social Sciences, Bar Ilan University, Ramat Gan, Israel; ^2^The Lester and Sally Entin Faculty of Humanities, The Jaime and Joan Constantiner School of Education, Tel Aviv University, Tel Aviv, Israel

**Keywords:** affix, spelling, development, morphology, word frequency

## Abstract

The current study examined the factors underlying native Hebrew speakers’ ability to learn homophonous affix spelling. It takes a novel view in investigating the effect of morpho-orthographic complexity of affix representation on the development of affix spelling across the school years. The role of five morpho-orthographic principles in homophonous affix letter spelling was studied: (i) morpho-orthographic transparency; (ii) affix letter prevalence; (iii) morpho-phonological competition; (iv) overtness of the phonological-orthographic link; and (v) phono-morpho-orthographic consistency. Taken together, these five principles of affix spelling constitute complexity metrics that pinpoint the loci of spelling challenge in homophonous Hebrew affixes. Study participants were 83 monolingual Hebrew-speaking students in four grade levels – 2nd, 4th, 7th, and 10th grades. The research instrument was a spelling task of 244 words containing affix letters in 57 morphological categories. The affixes appearing in the target words represented 56 different affix categories, covering all non-root morphological roles, both inflectional and derivational. While correct spelling increased across grade levels, a hierarchy emerged in interaction with grade level regarding these criteria: Younger spellers were mostly assisted by morpho-orthographic sites, morphological category frequency, and phonological transparency – while spelling in higher grade levels was more affected by morpho-orthographic prevalence. Thus, knowledge of how morphological roles are deployed in the orthography emerges as the most significant factor that affects learning to spell affix letters in Hebrew.

## Introduction

The development of spelling skills is essential for children’s literacy acquisition, as it increasingly promotes higher-order writing processes (Stage and Wagner, 1992; Graham and Santangelo, 2014). Much of the spelling literature primarily focused on how spellings are constructed from phonological forms (e.g., Barry and Seymour, 1988; Treiman et al., 2002; Treiman et al., 2015). This framework has been expanded in the last decades to a multi-faceted view of the combined roles of phonology and grammar in learning to spell. According to this view, several knowledge bases, including the phonological, orthographic, and morphological patterns inherent to words are involved in spelling acquisition from early childhood (Angelelli et al., 2014; Treiman and Kessler, 2014). The current investigation is at the interface of Hebrew phonology, morphology and orthography with cognitive factors of pattern detection and generalization, on the one hand, and psycholinguistic factors of transparency, frequency, and prevalence, on the other, across the school years. Specifically, we examine the effect of morpho-orthographic complexity on the path of acquisition of homophonous affix spelling in Hebrew-speaking spellers from 2nd to 10th grade. Complexity is expressed by five novel metrics: (i) morpho-orthographic transparency; (ii) affix letter prevalence; (iii) morpho-phonological competition; (iv) overtness of the phonological-orthographic link; and (v) phono-morpho-orthographic consistency.

### Theoretical Framework

This study arises from several theoretical assumptions, which provide the framework for the study and its hypotheses.

#### Usage-Based Learning

The psycholinguistic learning theory adopted in the current context is the Usage-Based approach, where learning is regarded as the result of discerning repeating patterns in the input, leading to the emergence of categories as the result of changes in the system (Diessel, 2019). Linguistic systematicity emerges from experience with individual usage events during exposure to spoken and written language, in a process that is graded, probabilistic, interactive, and context-sensitive, under constant pressure from changing linguistic input (Tomasello, 2003; Abbot-Smith and Tomasello, 2006; McCauley and Christiansen, 2019). Importantly, usage-based approaches emphasize the critical role of low-level (i.e., relatively specific) generalizations in learning, taking into account frequency factors and the similarity of the exemplar being learned to others already stored. Thus, spelling performance improves over many learning trials as morpho-lexical patterns are learned as generalizations over memories of words (Aguado-Orea and Pine, 2015; Rácz et al., 2015). The diachronic process whereby the consistent morphological spelling of English derivational suffixes arose from homophonous variants supports this view of usage-based development (Berg and Aronoff, 2017).

As the source of generalizations is the ambient (spoken and written) language, native language learners need to pay attention to the special typological features of the language being learned (Cysouw, 2005), such as a rich morphology (Ravid, 2012, 2019b), and the properties of the notational system being learned (Ravid and Tolchinsky, 2002; Sampson, 2016; Martinez-Adrian and Gallardo-Del-Puerto, 2017).

#### Spelling as a Source of Lexical Quality

A second theoretical assumption guiding the current study is that spelling knowledge is lexical in nature, that is, it is part of language users’ knowledge about words and patterns of similarities that link words together. Recent models of the mental lexicon regard it as a dynamic structure in which words constitute the prime lexical representations (Blevins, 2016). Words may be similar in sound (*doctor, document*), in meaning (*tall, high*), or in meaning-bearing structure (*high/height, document/documentary*). With time and frequent use, these links come to organize the mental lexicon by abstract representations of phonological, semantic or morphological similarity patterns (Clark, 2017), yielding a system of constructions that is capable of expressing meaning matched to form (Goldberg, 2006). Studies of spoken language acquisition underscore the role of statistical properties of the ambient language (such as type and token frequency, transparency, regularity, consistency, salience, and neighborhood density) in the process of language acquisition and development (MacRoy-Higgins et al., 2013; Nation, 2013; Ambridge et al., 2015). In learning to spell, learners would be looking for similar consistent and meaningful statistical patterns in the visual representation of word-internal units that they have mapped out for spoken language (Levin et al., 2001; Ravid, 2012; McCutchen and Stull, 2015; Northey et al., 2016; Treiman, 2018a; Treiman et al., 2019). Therefore, current thought in the developmental psycholinguistics of spelling is that its acquisition and consolidation constitute part of the lexical and grammatical knowledge that children accumulate across the school years (Treiman, 2017). Learning to spell is regarded as part of the acquisition of “lexical quality” in a particular language, and good spellers have qualitative lexical representations (Perfetti, 2007): The more a person knows about a word in terms of its lexical semantics, phonology, morphology, and syntax, the more “qualitative” its representation and retrieval. A stable orthographic representation (= correct spelling) is an important signal of a word’s lexical quality.

#### Language Typology, Orthographic Typology and Morphological Cues

A third theoretical assumption relates to the language type, the type of the orthography, and their relation to spelling acquisition. From the point of view of the language type, the organization of the lexicon in a given language has developmental implications. Children growing up in morphology-rich languages figure out early on that this is “where the action is” – where meanings and forms densely coalesce in language-specific ways (Berman, 1985). In learning to spell, these children would be looking for the very same categories and relationships in written language that they have mapped out for spoken language.

From the point of view of the orthographic type, we should note the relationship between orthography, phonology and morphology. In languages with alphabetical orthographies, the grapho-phonemic code expresses the crucial relationship between orthography and phonology, and learning to spell begins with cracking this code, creating pathways that delineate coarse-grained networks between phonological segments and graphemes that are adequate for reading (Goswami, 2002; Treiman, 2018b). However, in very few, if any, orthographies, is this initial knowledge adequate for the level of spelling, which requires finer-grained mappings and hence precision in selecting one grapheme over another (Holmes and Babauta, 2005). In fact, alphabetical orthographies often systematically express meanings or affix functions via written morphological units, and they may ignore finer morpho-phonological distinctions to express the meaningful generalizations of the morphological system (see a current summary in Sandra, 2018).

In many languages with alphabetical orthographies, morphology – the structural organization of meaning within the word – constitutes the architecture of hidden units mediating the complex and often opaque relationships between phonology and orthography. This is certainly true for Hebrew, where many semantic notions are expressed within the word, with major morphological systems organizing the lexicon and morpho-phonological alternations prevalent in it (Ravid, 2012, 2019a; Schwarzwald, 2002). But it is also true of languages with less dense morphological systematicity: A recent corpus study of written English across a millennium (750–1700 AD) traces the emergence of affix spelling from variegated beginnings to clear morphological marking of nouns, adjectives, and verbs – e.g., the –OUS adjectival suffix (Berg and Aronoff, 2017). The authors remark that “Crucially, this is information that the phonological system does not provide – it is a distinct feature of the writing system” (p. 45), in which the spelling distinguishes homophonous suffixes or word endings and allows readers to access lexical and syntactic information directly from the orthographic representations.

As written language represents morphological constructs distinctly from spoken language, often overriding or ignoring phonological constructs, the task of the learning speller would then be to discover and identify morphological categories in the orthography and link them up to spoken categories. In learning to spell morphological categories, the strength of a morphological pattern depends on the number of words that exhibit it, and on the degree to which it is evident in these words.

Affix spelling (e.g., past-tense *–ed*) has been the subject of a series of acquisitional studies on grade school children, showing how morphology increasingly participated in children’s spelling choices. Nunes et al. (1997) found that English-speaking first grade children learned the bi-morphemic nature of KISSED (first spelled KIST), and then overgeneralized this spelling (e.g., SOFED for SOFT), before reaching correct usage (Walker and Hauerwas, 2006). The effect of morphological learning was also apparent in the tendency to simplify consonant clusters more in mono-morphemic words (e.g., BRAND) than in bi-morphemic words (e.g., RAINED) (Treiman and Cassar, 1996). In the same way, Kandel et al. (2012) found that French-speaking children took longer to write at the morpheme boundaries of derived words. A similar route was found in a longitudinal study of Greek first graders (Chliounaki and Bryant, 2002) who were learning to spell the alternative orthographic forms of the vowels *o* and *e*: As children began to use the new spellings, they overgeneralized them to inappropriate environments, first in inflections and then in stems. Finally, correct spellings were restricted to appropriate contexts. Moreover, Nunes et al. (2006) report that correct spelling of -ED predicted performance on different tasks of morphological awareness even when controlling for age and IQ, showing that children’s morphological representations were enhanced as a result of learning to spell those morphemes. These studies testify to children’s emerging construal of morphemes in writing and the gradual establishment of morpho-graphic patterns. Importantly, it seems that children’s ability to process the word’s morphological structure assists them as they start to spell, interfacing with phonology, semantics and orthography (Breadmore and Deacon, 2019).

Against this background, the approach in the current study pinpoints three knowledge domains as necessary to spelling acquisition: (i) how phonological segments map onto graphemes; (ii) the specific properties of the orthographic system; and (iii) the nature of the morphological segments represented by the orthography. In order to acquire mature knowledge of an orthography, a learner has to be proficient in each of these different domains, to construct their cognitive representations so as be able to retrieve them at will, and to map this knowledge onto the specific orthography being learned (Kargl and Landerl, 2018). The goal of spelling is thus achieving a high-quality lexical fit with correct orthographic representation, which expresses morphological information (Desrochers et al., 2018). To this end, young spellers need to keep track of multiple co-occurrences of different units, monitoring the frequencies, regularities and consistent behavior of phonemes and morphemes in words, on the one hand, and how they are expressed in the specific orthographic patterns of their language, on the other.

### Hebrew Spelling: Orthography, Phonology, Morphology

The current study is a developmental investigation of how children learn to spell affix letters in Hebrew, a language with non-linear morphology, where discontinuous roots and prosodic patterns combine to form words. As an initial example, take ***l****i****m****e****d****/me****l****u****m****a****d****/****t****a****l****mi****d*** “taught/scholar/pupil,” three words that share the same root *l-m-d* meaning “learn” (in bold). The same root is inserted into three different pattern templates that provide vowels interspersed between root radicals and consonantal prefixes or suffixes. Relevantly, recent models of spelling representation (Dehaene et al., 2006) suggest that our brain is sensitive not only to adjacent but also to discontinuous letter combinations that need to be tracked, as in the case of Hebrew.

A major challenge in spelling Hebrew involves the non-transparent mapping of phonology to the orthography, that is, homophony. Extensive neutralizations (or mergers) of previously distinct phonemes have rendered Modern Hebrew phonology very different from its Classical counterparts (Bolozky, 1997; Ravid, 2005). Several sets of Classical consonants merged, resulting in the loss of historical phonological distinctions (Weinberg, 1966; Laufer and Condax, 1981; Schwarzwald, 2001). When phonological distinctions are no longer directly encoded in the orthography, homophony is entailed: a single phoneme can be spelled by more than one grapheme. In order to correctly spell a homophone (e.g., *t* by either ט or ט), knowing the morphological role it serves in the word is critical. As root letters, homophones are extremely challenging, with high type frequency and low token frequency: All 22 letters participate in about 1,500 different roots (Ravid et al., 2016), with the Zipfian frequency typical of lexical elements, so that many repeated occurrences of the same root are necessary for its spelling. However, natural language texts are not “saturated” by lexical units (words and roots), that is, words and roots do not occur repeatedly in the same corpus (Ackerman and Malouf, 2013). In addition, the choice of correct root spelling is conditioned by a complex set of characteristics including root radical position, letter frequency, and morpho-phonological considerations. Therefore, the acquisition of root spelling is a long and arduous process (Ravid, 2001, 2005, 2012).

#### Affix Spelling in Hebrew

In contrast, affix spelling, which is the focus of the current study, is generally less challenging than root spelling, as most affixes have lower type and higher token frequencies, coupled with higher morpho-orthographic transparency, than roots (Ravid, 2001). Only 11 of the 22 alphabet letters serve in affix letters, and they stand for about 20 morphological roles, both derivational and inflectional. Texts are much more saturated by affix than by root morphemes. Thus, learning to spell affix letters is aided by the low type frequency of affix letters, on the one hand, and their very high token frequency as grammatical morphemes with various affixes, on the other. Most importantly, the phonological-orthographic complexity of homophones is reduced in affix spelling, as in most cases, only one of the two possible graphemes serves as an affix letter. For example, of the two letters ת, ט representing *t*, only ת represents an affix. The same is true of the two letters כ, ק representing *k*, and the two letters כ, ח representing *x*, respectively: ק and ח do not have affix roles. Therefore, a large part of the homophonous challenge to spelling affix letters disappears (Gillis and Ravid, 2006).

A major challenge for affix spelling in Hebrew is identifying affixes as such: spellers need to know whether the homophone has a root or affix role, as this will determine the path in spelling. As a root segment, both spelling options are viable, and thus challenging as homophones; but as an affix letter, homophony is in most cases no longer a problem. The orthographic structure of the Hebrew word is helpful in the identification of letters as roots or affixes: root letters typically congregate in the center of the written Hebrew word, whereas affix letters take peripheral positions in the outer envelope of the word. For example, the written string ובמכתבים “and-in-the-letter-s,” the bolded letters at both sides of the root כתב “write” respectively, from right to left, represent affixal roles of conjunction, preposition, pattern prefix and plural suffix. And in the string לכשתבואנה “by the time-they (feminine)-will-arrive,” the bolded affix letters, respectively, represent the roles of time, future tense, third person, feminine, and plural. The small number of affixes (low type frequency), their ubiquity in the language (high token frequency), and their distinct peripheral positions all serve as reliable morphological pointers to affix morphology. Therefore, identifying the morphological role of the homophonous letter as an affix versus root letter is critical in achieving correct spelling (Ravid, 2012).

However, not all affixes are easy to identify in their non-root roles, as the boundaries between root and affix sites might be blurred. This can happen, for example, in words with irregular roots such as *t****o****’é****l****et* תועלת “benefit,” where the root (bolded in transcription and in Hebrew script) is not entirely consonantal, so that the first ת might be interpreted as a root letter. There are other factors that might stand in the way of a successful mapping of the morphology-phonology-orthography link, which promotes correct spelling. Frequency and coherence (= consistency) of letter, word and category can hinder or facilitate affix identification and spelling, especially in specific sites. Thus, for example, consonantal *v* is more likely to be linked to ו as an affix at the beginning of the word (the conjunction *ve*-), and less to ו at the end of a word, where it has few roles, e.g., representing an allomorph of the 3rd person possessive in *–iv.*

Previous studies have suggested that children learning to spell consider the morphological regularities of their orthography, and that they might also use large-sized processing units in spelling (Angelelli et al., 2014; Treiman, 2017). However, to the best of our knowledge, no study has produced a metric to classify and quantify the complexity of affix spellings. Previous studies have established that frequency seems to play a major role in the development of affix spelling because children’s spelling accuracy increases as they progress in age (e.g., Treiman et al., 1993). For example, English-speaking and French-speaking children demonstrated implicit learning of the morphological patterns in their orthography (Cassar and Treiman, 1997; Pacton et al., 2001, 2002), leading them to prefer the more frequent orthographic spelling patterns.

Moreover, recent studies have pointed to the characteristics of the alphabetic writing system as a major factor that influences spellers’ sensitivity to morphology and not just phonology when determining which spelling alternative is correct (Angelelli et al., 2014; Treiman, 2017). A study including French-speaking first, second, and third graders that examined the acquisition of silent-letter endings (Sénéchal et al., 2016) demonstrated that the absence of phonological cues resulted in children making more errors in pseudoword spelling with silent-letter endings. Another study with Arabic-speaking high school students revealed that 10th graders were still making mistakes when affix letters were interdigitated within root letters, indicating that when affixation modifies the morphological structure of the word, choosing the familiar letter string is still a dominant strategy (Oren, 2001). Taken together, it appears that using a familiar orthographic form is a common strategy of spelling production, even in skilled spellers (Treiman et al., 2015).

### Current Study

This current Hebrew study differs from previous studies on the same topic in several respects (Treiman et al., 1993; Treiman and Cassar, 1997; Pacton et al., 2001, 2002; Angelelli et al., 2014; Sénéchal et al., 2016; Treiman, 2017). First, from a typological perspective, the current study examines spelling acquisition as a specific morphological knowledge domain constrained by the morpho-orthographic behavior of affix letters. Second, from a developmental perspective, spelling achievement is examined across the school years as increasingly complex spelling patterns are overcome in learning. A final perspective is gained through the classification and mapping of affix spelling complexity according to five morpho-orthographic principles, as elaborated below in the “Materials and Methods” section.

The current study takes a novel view in investigating the effect of morpho-orthographic complexity of affix representation on the development of Hebrew spelling across the school years. We specifically examined the role of five morpho-orthographic principles in homophonous affix letter spelling: (i) morpho-orthographic transparency; (ii) affix letter prevalence; (iii) morpho-phonological competition; (iv) overtness of the phonological-orthographic link; and (v) phono-morpho-orthographic consistency (see a detailed presentation in the “Materials and Methods” section). Taken together, these five principles of affix spelling constitute complexity metrics that pinpoint the loci of spelling challenges in homophonous Hebrew affixes. Their examination in the current study provides a fine-grained depiction of the development of affix spelling and the factors that determine the sequence and pace of its acquisition. We hypothesized that word frequency and the morpho-orthographic metrics of affix spelling constitute two separate factors that independently predict different spelling skills across development. To investigate this hypothesis, the Affix Letter Spelling Task, representing all affixes and all of their morphological roles in Hebrew, was administered to the study participants, as delineated below. Given the literature review, we predicted higher scores on high-frequency words, words with transparent demarcation of root from affix envelope, prevalent affixes, affixes without morphological competition, affixes expressing overt phonological-orthographic links, and affixes with phono-morpho-orthographic consistency.

## Materials and Methods

### Participants

Study participants were 83 monolingual Hebrew-speaking students in four grade levels – 2nd, 4th, 7th, and 10th grades (39 boys and 44 girls). No significant difference was found in the gender distribution between the four grade levels *χ^2^*(3) = 1.49, *p* = 0.684. Participants were typically developing readers selected according to the following criteria: (i) normal performance on a non-verbal general intelligence test (Wechsler, 1991); (ii) performance on a standard vocabulary test (Wechsler, 1999); and (iii) normal reading speed and accuracy on a standard reading test (Schiff et al., 2008). Table 1 shows the background characteristics of the participants (gender and age) and their performances on the background tests. Results showed that participants’ non-verbal intelligence, vocabulary, and reading accuracy and fluency scores were within the normal range. Furthermore, none of the participants had any hearing impairment, attention deficit disorder nor a history of neurological or emotional disorder, as reported by clinicians, educational professionals and the adult participants themselves.

**TABLE 1:** The background characteristics of the participants (gender and age) and their performances on the vocabulary and reading tests.

	Second	Fourth	Seventh	Tenth	Statistical differences
	(1)	(2)	(3)	(4)	
Gender (Boys/Girls)	12/11	8/14	10/11	9/8	*χ^2^*(3) = 1.49, *p* = 0.684
Age	7.48 (0.49)	9.55 (0.41)	12.64 (0.45)	15.62 (0.49)	*F*(3,79) = 1196.36, *p* = 0.000, η_*p*_^2^ = 0.98 *Scheffe: 1* < *2* < *3* < *4*
Vocabulary^1^	10.26 (1.42)	10.14 (1.88)	10.29 (1.35)	10.35 (1.87)	*F*(3,79) = 0.06, *p* = *0.980*, η_*p*_^2^ = 0.00
Matrix^1^	10.57 (1.38)	10.50 (1.54)	11.24 (1.30)	11.00 (1.97)	*F*(3,79) = 1.12, *p* = 0.346, η_*p*_^2^ = 0.04
Reading speed^2^	43.17 (10.73)	52.86 (7.51)	74.52 (9.95)	76.59 (8.69)	*F*(3,79) = 63.77, *p* = 0.000, η_*p*_^2^ = 0.71 *Scheffe: 1* < *2* < *3* = *4*
Reading accuracy^2^	77.91 (9.40)	87.18 (7.08)	96.19 (4.62)	102.06 (4.92)	*F*(3,79) = 47.06, *p* = 0.000, η_*p*_^2^ = 0.64 *Scheffe: 1* < *2* < *3* = *4*

*^1^Normal performance – all the performance ranged between 7 and 13 (M = 10.00, SD = 1.50); ^2^The reading speed and accuracy are in an appropriate level regarding the participants age.*

Participants were selected from public primary schools and high school in the greater Tel Aviv area in Israel. The Tel Aviv schools chosen for the study are located in the center as well as the northern part of the city. The study was conducted according to the principles of the Helsinki Declaration and was approved by the Ministry of Education and the Institutional Review Board at Bar Ilan University. Parents were informed of the screening activities and had to approve their child’s participation. All data concerning individual performances were analyzed strictly for research purposes.

### Materials

#### The Screening Tests

Non-verbal intelligence was assessed by the WASI Matrix Reasoning subtest (Wechsler, 1999). This task requires participants to choose an item from the bottom of the figure that would complete the pattern at the top. The maximum raw score is 60. Test reliability coefficient is 0.96.

Vocabulary was also assessed by the WASI Matrix Reasoning subtest (Wechsler, 1999). The vocabulary subset consists of four picture items and 38 word items.

The word reading accuracy test required participants to read aloud a list containing 112 non-vowelized words. Scores ranged from 0 to 112, reflecting the number of correct answers given, with higher scores indicating higher reading accuracy. In the word reading fluency test, participants read aloud as many words as possible in 45 s from a list containing 104 words (Schiff et al., 2008). The wordlist used in the fluency test differed from the words used in the accuracy test. Scores ranged from 0 to 104, reflecting the number of accurate words the participant read in 45 s, with higher scores indicating higher reading fluency. Words in both tests increased in difficulty.

#### The Affix Letter Spelling Task

The research instrument was a spelling-to-dictation task, which consisted of 224 words, each containing one homophonous affix letter (Schiff and Levie, 2017). The affixes appearing in the target words represented 56 different affix categories (four words per affix category), covering all of the function (non-root) morphological roles of Hebrew affix letters, both inflectional and derivational (Ravid, 2012). For example, the prefixal conjunction *ve* “and” spelled ו constituted one category, the tense/person prefix *t-* spelled ת constituted another, the nominal pattern suffix *t-* spelled ת constituted yet another category, and the suffixal *-xa* indicating second person masculine was a fourth affix category.

Half of the words (112) were of high frequency and half (112) were of low frequency. In order to validate the classification of the division of the words in the spelling task according to their level of frequency in the language (low frequency, high frequency), an initial list of 228 words was given to ten judges, experts in the field of language and Hebrew linguistics. Each judge was requested to rank the level of frequency of the word on a scale of 1 (the word is not frequently used in the language) to 5 (the word is frequently used in the language). Words that were ranked by all ten judges as having frequency levels of 1 or 2 in the language were defined as non-frequent words, while words that were ranked by all ten judges as having frequency levels of 4 or 5 were defined as frequent words. Four words were removed from the final test administered to the participants of the study, due to lack of consent among the judges with regards to their frequent use in the language.

### Procedure

Words in the spelling test were randomized and administered in a spelling-to-dictation task. The dictation task was administered orally and individually, preceded by three examples. Each target word was presented in the context of a short sentence to assure clarity of meaning and eliminate possible ambiguity. The examiner read aloud each target word in its sentential context in a neutral tone without emphasizing the presence of possible orthographic difficulties. Participants were instructed to write only the target word, which was repeated at the beginning and end of each sentence (Gillis and Ravid, 2006). For example: *mishkéfet, yesh la-yéled mishkéfet*; *tixtevu mishkéfet* “goggles, the boy has goggles; please write: goggles.” To ensure that the children had correctly perceived the target words, the examiner asked them to repeat each one before they wrote it down. No feedback was provided on the correctness of the written response. Pauses were allowed if requested. Spontaneous corrections were accepted.

### Coding

Two variables were taken into consideration in coding the task affixes – the frequency of the word in which the target affix appeared and the five morpho-orthographic principles of homophonous affix spelling, serving as criteria for evaluating affix complexity. Thus, each affix on the test was assigned binary values regarding each of the five criteria, as explained, illustrated and motivated below.

(1) The first criterion was *the transparency of the affix envelope*, i.e., the degree to which it is possible to demarcate the central root morpheme from the affixal periphery. For example, in transparently structured words such as תרדמה *ta****rd****e****m****a* “slumber,” it is easy to perceive the affix letters ה, ת (signifying pattern prefix and suffix) in the margins of the word, clearly demarcated from the regular, consonantal root morpheme in the center of the word. However, the root in *t****o****’é****l****et* תועלת “benefit” (in bold in the transcription and in the Hebrew script) is irregular, partially non-consonantal, so that the ו at its beginning can be confused with marking a pattern vowel. This obscures the construal of the word’s prefixal ת and suffixal ת as affixes: the first ת can be easily interpreted as a root letter, as in the superficially similar word *toféret* תופרת “seamstress.” The binary value for this criterion was either clearly demarcated or opaque. When the affix envelope is clearly demarcated, it is easier to identify homophonous letters as belonging to it and thus to reduce homophony complexity. We expected homophonous letters in clearly demarcated envelopes to be spelled more correctly.
(2) *Affix letter prevalence*, that is, the frequency of the letter in its morphological and orthographic roles, represents its category size. This notion reflects to what extent the number of morphological affix roles the letter represents as well as their variety and prevalence (Ravid, 2012). Thus, a letter with many affix roles is likely to have many occurrences in written Hebrew, which would strengthen not only the role of the letter as affix but also the environments where it is likely to appear (Ambridge et al., 2015). For example, ת (consistently pronounced *t*), which appears in both prefix and suffix positions, has 11 morphological roles, not only signifying feminine gender and second person in various contexts, but also participating in many derivational roles (Ravid, 2019a). In contrast, כ has only two affix roles, both inflectional, which are obscured by the fact that כ represents both the stop *k* and the spirant *x*, i.e., is not phonologically stable; with further constraints on its occurrence in these phonological roles as prefix or suffix. The morpho-orthographic prevalence of letters in the current study was based on the analyses in Ravid (2012, 2019a). It was further assessed by four language and spelling experts, who assessed the category size for each affix letter in each of the task words on a scale from 1 to 5, with a high value of Cronbach’s alpha (0.90). The binary value for this criterion was either prevalent or non-prevalent. We thus expected homophonous letters with high prevalence to be spelled more correctly.
(3) A third criterion took into account the existence of morphological “enemies,” i.e., *internal morpho-phonological competitors*. This criterion resonates the main pathway to correct spelling in the identification of homophonous letters in their affix roles, i.e., lack of graphemic competitors for the same phonological segment. However, the identification of an affix letter as such (that is, not a root letter) is a necessary, but not a sufficient condition for correct affix spelling even in clearly demarcated environments. The sufficient condition is the absence of competitors in the *same* affix role. This condition becomes necessary in the spelling of ה *h*, and י *y*, which both serve as tense (past and future tense, tense, respectively) prefixes in specific verb morphology environments. For example, 3rd person singular ***h****itkadem* “advanced” התקדם and ***y****itkadem* “will advance” תקדם differ only in the first letter of the prefix, which is extremely similar phonologically and also shares the same inflectional role (tense-person). Such competition *within the affix category* reduces spellers’ ability to differentiate between the two homophones. The binary value for this criterion was either the presence or absence of internal competitors. We expected homophonous segments with internal morpho-phonological competitors to increase the complexity of the spelling task and thus to reduce success scores, especially in younger spellers.
(4) A fourth criterion concerned *the overtness of the phonological-orthographic* link. In most cases of Hebrew spelling, phonological information is directly linked to the orthography (even though they may not be consistently linked in the case of homophony). Thus, in most cases a letter represents a phonological segment. However, there are cases of *covert* phonology, where the orthographic segment does not represent a phonological unit, but rather, and only, a morphological unit. For example, the possessive suffix *-av* “his” יו - is spelled with י *y* which is not directly linked to any phonological segment normally related to י. The binary value for this criterion was either overt or covert phonology. We expected covert phonology to increase the complexity of the spelling task and thus reduce success scores, especially in younger spellers.
(5) A fifth and last criterion was *phono-morpho-orthographic consistency*, relating to the degree of consistency in spelling patterns that spellers can adhere to as a generalization. One such spelling pattern is the prevalent link between a final feminine -*a* being universally spelled by ה, as in the feminine noun, adjective and verb *malk****a*** מלכה “queen,” *sgur****a*** סגורה “closed,” and *hisbir****a*** הסבירה “explained,” respectively. This link and similar spelling patterns generally point to the representation of final vowels by one of the אהוי vowel-marking letters (Ravid, 2012). The generalization that is elicited from these highly frequent spelling patterns is that open syllables at the end of a word should be “closed” in writing by one of the אהוי letters, especially ה. When this generalization is violated, as in *katávta* כתבת “you, masculine wrote,” it is very difficult to overcome the tendency to add a final closing letter. The binary value for this criterion was either conforming or violating phono-morpho-orthographic consistency. We expected the violation of consistent spelling patterns to increase spelling complexity, and to be acquired later on.

## Results

Homophonous affix letters in the study were each assigned five binary values corresponding to the five morphological criteria as described above under Coding. Before examining the study questions and hypotheses, we conducted Shapiro–Wilk tests in order to examine whether the spelling scores were normally distributed for each of the frequency values of each word (non-frequent words, frequent words), grades, and for each of the five criteria. Some of the success scores were not normally distributed. Therefore, we examined the study questions and hypotheses by conducting both parametric and non-parametric tests. The non-parametric analyses findings matched the findings of the parametric analyses. Therefore, we present the findings of the ANOVA’s analyses, instead of using non-parametric analyses – Wilcoxon tests. In the current study multiple hypotheses were tested among a small sample size. In these cases, the chance of observing a rare event increases, and the risk of making type I errors increases. In order to decrease this risk in cases where the two or three-way interactions were significant, Bonferroni correction was used.

In order to examine the success scores of homophonous affix spelling by word frequency, grade and each of the five study criteria, five 4 × 2 × 2 repeated measures analyses of variance (ANOVA) were conducted, with grade (second, fourth, seventh, tenth) as the between-subject variable, and word frequency (non-frequent words, frequent words) and the binary attribute of each criterion (no, yes) as the within-subject variables. It should be noted that prior to the examining the research questions, we looked at potential gender differences in the spelling scores on the frequency values of each word, grade and for each of the five criteria. No significant differences between males and females were found in the spelling scores (*t*-values ranged between 0.02 and 1.93 and *p*-values ranged between 0.077 and 0.986). Therefore, the gender of the participants was not used as another between subject-variable in the repeated measures analyses.

### Transparency of the Affix Envelope

The main effects of word frequency and transparency of affix envelope were significant [*F*(1,79) = 32.82, *p* = 0.000, η_*p*_^2^ = 0.29 and *F*(1,79) = 367.05, *p* = 0.000, η_*p*_^2^ = 0.82, respectively], indicating higher spelling scores of frequent words and of affix letters in demarcated envelopes. Furthermore, the main effect of grade was also significant, *F*(3,79) = 46.26, *p* = 0.000, η_*p*_^2^ = 0.64. Scheffe *post hoc* analysis indicated that spelling scores increased with age and schooling. No significant differences were found in the spelling scores between seventh and tenth grade students (*p* = 0.299).

The two-way interaction of grade and transparency of affix envelope was significant, *F*(3,79) = 25.94, *p* = 0.000, η_*p*_^2^ = 0.50. Bonferroni analyses indicated that the spelling scores of affix letters in demarcated envelopes were significantly greater than in the non-demarcated envelopes in all grades (*ps* = 0.000). The effect size decreased as the age of the student increased (η_*p*_^2^ = 0.93, η_*p*_^2^ = 0.82, η_*p*_^2^ = 0.69, and η_*p*_^2^ = 0.70 for the second, fourth, seventh, and tenth grades).

The two way interaction of word frequency and transparency of affix envelope was also significant, *F*(1,79) = 4.94, *p* = 0.029, η_*p*_^2^ = 0.06. Bonferroni analyses indicated that the spelling scores of affix letters in demarcated envelopes were significantly greater than in non-demarcated envelopes in both frequent and non-frequent words (*ps* = 0.000). The effect size was greater in non-frequent words compared to frequent words (η_*p*_^2^ = 0.75 and η_*p*_^2^ = 0.60, respectively).

Finally, the two way interaction of grade and word frequency and the three way interaction were not significant [*F*(3,79) = 1.74, *p* = 0.166, η_*p*_^2^ = 0.06 and *F*(3,79) = 0.99, *p* = 0.403, η_*p*_^2^ = 0.04, respectively] (see Table 2).

**TABLE 2:** Means (and SD) of the success scores (%) of spelling function by word frequency, grade and demarcated function envelope.

		Non-demarcated envelope		Demarcated envelope	
Word frequency	Grade	Mean	SD	Mean	SD
Non-frequent words	Second	42.75%	13.13	81.80%	8.13
	Fourth	59.85%	14.00	88.13%	7.75
	Seventh	75.79%	15.57	95.14%	2.89
	Tenth	85.78%	7.07	98.73%	0.79
Frequent words	Second	46.38%	19.43	86.02%	8.12
	Fourth	70.45%	21.32	92.35%	6.31
	Seventh	83.33%	11.79	97.60%	1.99
	Tenth	89.71%	10.00	99.28%	0.36

### Affix Letter Prevalence

The main effects of word frequency and affix letter prevalence were significant [*F*(1,79) = 47.98, *p* = 0.000, η_*p*_^2^ = 0.38 and *F*(1,79) = 332.68, *p* = 0.000, η_*p*_^2^ = 0.81, respectively], indicating higher spelling scores of frequent words as well as when the letter is prevalent in its morphological roles. Furthermore, the main effect of grade was also significant, *F*(3,79) = 45.10, *p* = 0.000, η_*p*_^2^ = 0.63. Scheffe *post hoc* analysis indicated that spelling scores increased with age and schooling. No significant differences were found in the spelling scores between the seventh and the tenth grade students (*p* = 0.49).

**FIGURE 1:**
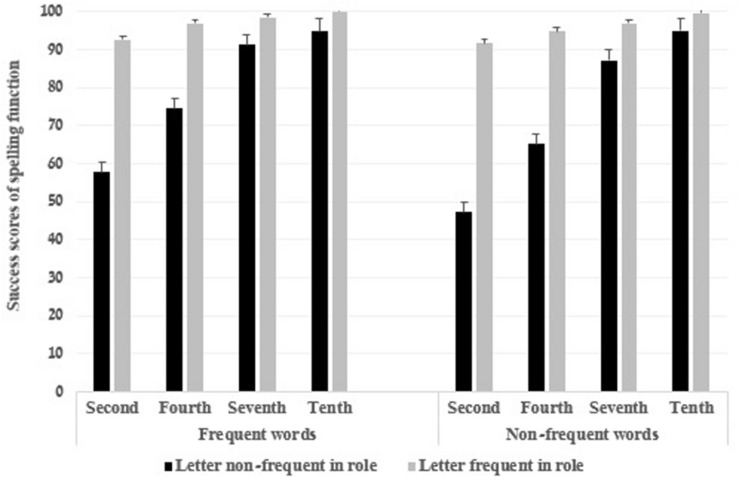
The success scores (%) of spelling function by word frequency, grade, and letter frequency in its morphological role.

All the two-way interactions were significant [grade and word frequency: *F*(3,79) = 6.53, *p* = 0.001, η_*p*_^2^ = 0.20, grade and affix letter prevalence; *F*(3,79) = 55.94, *p* = 0.000, η_*p*_^2^ = 0.68, word frequency and affix letter prevalence: *F*(1,79) = 30.27, *p* = 0.000, η_*p*_^2^ = 0.28]. Finally, the three-way interaction was significant *F*(3,79) = 6.87, *p* = 0.000, η_*p*_^2^ = 0.21. Bonferroni analyses indicated that the spelling scores of affix letters when the letter is prevalent in its morphological roles were significantly greater than when the letter is not, in the second, fourth, and seventh grades (*ps* = 0.000) but not in the tenth grade (*ps* > 0.05). These results were found in both frequent and non-frequent words. The effect sizes were greater in non-frequent words compared to frequent words (Non-frequent words: η_*p*_^2^ = 0.96, η_*p*_^2^ = 0.80, η_*p*_^2^ = 0.70 for the second, fourth, and seventh grades and η_*p*_^2^ = 0.84, η_*p*_^2^ = 0.75, η_*p*_^2^ = 0.63 for the frequent words) (see Figure 1).

### Morpho-Phonological Competition

The main effects of word frequency and morpho-phonological competition were significant [*F*(1,79) = 46.64, *p* = 0.000, η_*p*_^2^ = 0.37 and *F*(1,79) = 116.14, *p* = 0.000, η_*p*_^2^ = 0.59, respectively], indicating higher spelling scores of frequent words and in cases of no competitors. Furthermore, the main effect of grade was also significant, *F*(3,79) = 37.58, *p* = 0.000, η_*p*_^2^ = 0.59. Scheffe *post hoc* analysis indicated that the spelling scores increased with age and schooling. No significant differences were found in the spelling scores between the seventh and the tenth grade students (*p* = 0.713).

The two-way interaction of grade and word frequency was significant, *F*(3,79) = 4.55, *p* = 0.005, η_*p*_^2^ = 0.15. Bonferroni analyses indicated that spelling scores of frequent words were significantly higher than non-frequent words among the second, fourth and seventh grades (*ps* = 0.000), but not in the tenth grade (*p* = 0.756).

The two-way interaction of grade and morpho-phonological competition was significant, *F*(3,79) = 28.41, *p* = 0.000, η_*p*_^2^ = 0.52. Bonferroni analyses indicated that the spelling scores of affix letters in cases of no competitors were significantly greater than in cases with competitors, in the second, fourth, and seventh grades, but not in the tenth grade (*p* = 0.505).

The two way interaction of word frequency and morpho-phonological competition was also significant, *F*(1,79) = 7.69, *p* = 0.007, η_*p*_^2^ = 0.09. Bonferroni analyses indicated that the spelling scores of affix letters in cases of no competitors were significantly greater than in cases of no competitors in both frequent and non-frequent words (*ps* = 0.000). The effect size was greater in non-frequent words compared to frequent words (η_*p*_^2^ = 0.46 and η_*p*_^2^ = 0.43, respectively).

Finally, the three way interaction was not significant, *F*(3,79) = 1.26, *p* = 0.295, η_*p*_^2^ = 0.05 (see Table 3).

**TABLE 3:** Means (and SD) of the success scores (%) of spelling function by word frequency, grade and phonological and morphological competition.

		No-competitors		Competitors	
Word frequency	Grade	Mean	SD	Mean	SD
Non-frequent words	Second	90.15%	2.64	62.71%	15.48
	Fourth	93.54%	4.39	78.94%	14.87
	Seventh	96.73%	2.59	91.56%	4.86
	Tenth	98.30%	1.21	96.84%	2.62
Frequent words	Second	92.58%	3.60	66.69%	16.28
	Fourth	95.68%	4.05	83.32%	13.91
	Seventh	98.08%	1.47	94.30%	3.21
	Tenth	98.64%	1.36	96.94%	2.01

### Overtness of the Phonological-Orthographic Link

The main effects of word frequency and phonological overtness were significant [*F*(1,79) = 29.60, *p* = 0.000, η_*p*_^2^ = 0.27 and *F*(1,79) = 307.79, *p* = 0.000, η_*p*_^2^ = 0.80, respectively], indicating higher spelling scores of frequent words and with overt phonology. Furthermore, the main effect of grade was also significant, *F*(3,79) = 52.81, *p* = 0.000, η_*p*_^2^ = 0.67. Scheffe *post hoc* analysis indicated that the spelling scores increased with age and schooling. No significant differences were found in the spelling scores between the seventh and the tenth grade students (*p* = 0.616).

The two-way interaction of grade and word frequency was significant, *F*(3,79) = 4.11, *p* = 0.009, η_*p*_^2^ = 0.14. Bonferroni analyses indicated that spelling scores of frequent words significantly higher than non-frequent words among the second and the fourth grades (*ps* = 0.000), but not in the seventh and tenth grade (*p* = 0.075 and *p* = 0.773, respectively).

The two-way interaction of grade and phonological overtness was significant, *F*(3,79) = 39.80, *p* = 0.000, η_*p*_^2^ = 0.60. Bonferroni analyses indicated that the spelling scores of affix letters with overt phonology were significantly greater than with covert phonology in all grades (*ps* = 0.000). The effect size decreased as the age of the students increased (η_*p*_^2^ = 0.94, η_*p*_^2^ = 0.71, η_*p*_^2^ = 0.64, and η_*p*_^2^ = 0.59 for the second, fourth, seventh, and tenth grades).

The two way interaction of word frequency and phonological overtness was also significant, *F*(1,79) = 12.71, *p* = 0.001, η_*p*_^2^ = 0.14. Bonferroni analyses indicated that the spelling scores of affix letters with overt phonology were significantly greater than with covert phonology in both frequent and non-frequent words (*ps* = 0.000). The effect size was greater in non-frequent words compared to frequent words (η_*p*_^2^ = 0.64 and η_*p*_^2^ = 0.51, respectively). Finally, the three way interaction was not significant, *F*(3,79) = 2.34, *p* = 0.079, η_*p*_^2^ = 0.08 (see Table 4).

**TABLE 4:** Means (and SD) of the success scores (%) of spelling function by word frequency, grade and covert phonology.

		Covert		Overt	
Word frequency	Grade	Mean	SD	Mean	SD
Non-frequent words	Second	23.67%	16.85	82.76%	8.06
	Fourth	57.07%	20.66	88.33%	7.68
	Seventh	79.37%	11.80	95.17%	3.20
	Tenth	86.93%	10.57	99.08%	1.09
Frequent words	Second	42.51%	27.56	85.43%	7.83
	Fourth	70.71%	24.62	91.87%	6.45
	Seventh	85.19%	12.34	97.18%	2.12
	Tenth	88.24%	12.71	99.17%	0.45

### Phono-Morpho-Orthographic Consistency

The main effects of word frequency and phono-morpho-orthographic consistency were significant [*F*(1,79) = 37.57, *p* = 0.000, η_*p*_^2^ = 0.32 and *F*(1,79) = 43.15, *p* = 0.000, η_*p*_^2^ = 0.35, respectively], indicating higher spelling scores of frequent words and letters consistently following a generalization. Furthermore, the main effect of grade was also significant, *F*(3,79) = 33.79, *p* = 0.000, η_*p*_^2^ = 0.56. Scheffe *post hoc* analysis indicated that the spelling scores increased with age and schooling. No significant differences were found in the spelling scores between the seventh and the tenth grade students (*p* = 0.867).

**FIGURE 2:**
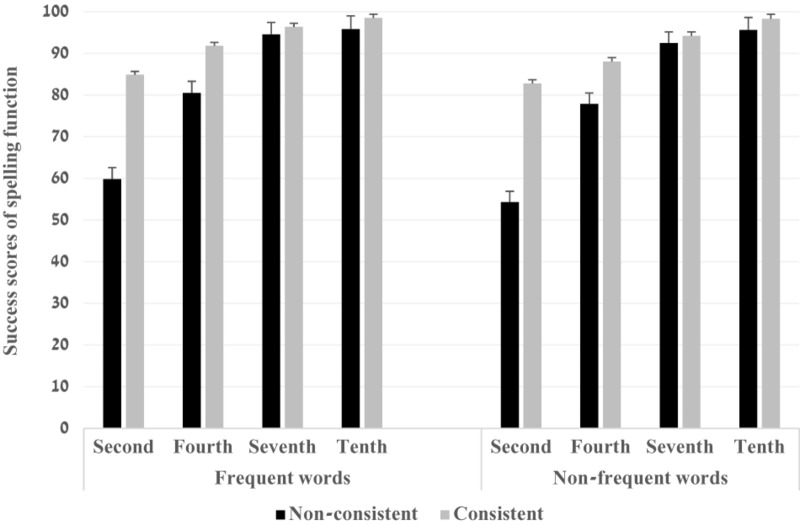
The success scores (%) of spelling function by word frequency, grade and orthographic consistency.

**FIGURE 3:**
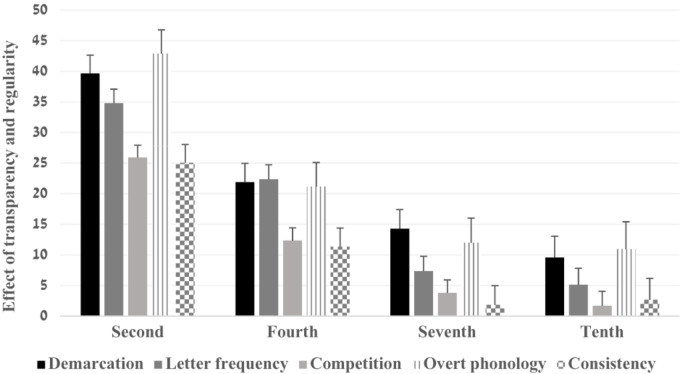
The effect of transparency and regularity by word frequency, grade and morphological categories in frequent words.

**FIGURE 4:**
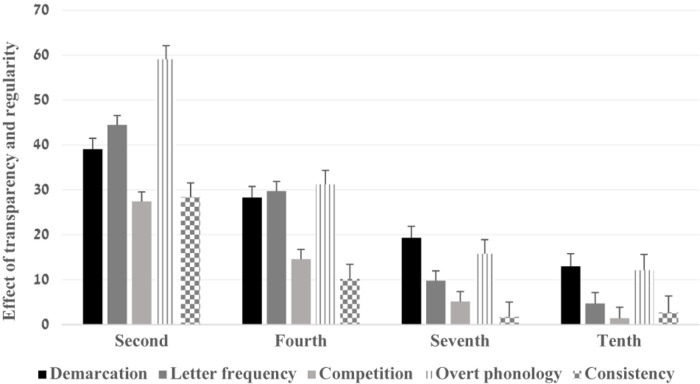
The effect of transparency and regularity by word frequency, grade and morphological categories in non-frequent words.

The two-way interactions of grade and word frequency, *F*(3,79) = 4.03, *p* = 0.010, η_*p*_^2^ = 0.13 and grade and phono-morpho-orthographic consistency, *F*(3,79) = 2.96, *p* = 0.000, η_*p*_^2^ = 0.10 were significant. The interaction of word frequency and phono-morpho-orthographic consistency was not significant, *F*(1,79) = 0.82, *p* = 0.367, η_*p*_^2^ = 0.01. Finally, the three-way interaction was significant *F*(3,79) = 2.96, *p* = 0.037, η_*p*_^2^ = 0.10. Bonferroni analyses indicated that the spelling scores of consistent affix letters were significantly greater than when the letter violated a generalization, in the second and fourth grades (*ps* = 0.000) but not in the seventh and tenth grade (*ps* > 0.05) (Figure 2). These results were found in both frequent and non-frequent words. The effect sizes were greater in non-frequent words compared to frequent words (Non-frequent words: η_*p*_^2^ = 0.64, η_*p*_^2^ = 0.29 for the second and fourth grades and η_*p*_^2^ = 0.57, η_*p*_^2^ = 0.41 for the frequent words) (see Figures 3, 4).

### Spelling Performance Across Morpho-Orthographic Criteria, Age and Frequency

The discrepancy between the binary values of each measure represents to what extent it results in higher success scores on spelling homophonous affix letters. In order to examine the differences in the discrepancy between the binary values of each morpho-orthographic principle of homophonous affix spelling by word frequency, grade and the five morphological categories, three-way (4 × 2 × 5) repeated measures analyses of variance (ANOVA) were conducted. The between-subject variable was grade; word frequency and affix regularity, transparency and consistency (RTC), as expressed by the five criteria, were the within-subject variables. The dependent variable was the level of discrepancy between the binary values that indicated the effects of affix RTC as expressed by the five criteria.

The main effect of word frequency was significant, *F*(1,79) = 20.99, *p* = 0.000, η_*p*_^2^ = 0.21, indicating a stronger effect of affix RTC in non-frequent words. The main effects of affix RTC was significant, *F*(4,76) = 23.47, *p* = 0.000, η_*p*_^2^ = 0.55. Bonferroni analysis indicated that transparent affix envelope, affix letter prevalence and overt phonology were more diagnostic than morpho-phonological competition and phono-morpho-orthographic consistency (*ps* = 0.000). Furthermore, the main effect of grade was also significant, *F*(3,79) = 55.09, *p* = 0.000, η_*p*_^2^ = 0.68. Scheffe *post hoc* analysis indicated that the spelling scores increased with age and schooling. No significant differences were found in the spelling scores between the seventh and the tenth grade students (*p* = 0.867).

The two-way interactions of grade and affix RTC *F*(12,201) = 4.10, *p* = 0.000, η_*p*_^2^ = 0.17 and word frequency and affix RTC, *F*(4,76) = 6.09, *p* = 0.000, η_*p*_^2^ = 0.24 were significant. The interaction of grade and word frequency was not significant, *F*(3,79) = 2.23, *p* = 0.092, η_*p*_^2^ = 0.08. Finally, the three-way interaction was significant *F*(12,201) = 3.97, *p* = 0.000, η_*p*_^2^ = 0.17. Bonferroni analyses indicated that transparent affix envelope, affix letter prevalence and overt phonology were more diagnostic than morpho-phonological competition and phono-morpho-orthographic consistency among the second and the fourth grades students (*ps* < 0.05). No significant differences were found between the morpho-phonological competition and phono-morpho-orthographic consistency criteria (*ps* = 0.99). In the seventh and in the tenth grades, affix envelope transparency was more diagnostic than competition. No significant differences were found between affix letter prevalence, phonological overtness and phono-morpho-orthographic consistency (*ps* > 0.05).

## Discussion

Selecting among the alternative spellings of a phoneme can be a challenge for spellers. Learning to spell in any alphabetical writing system requires understanding how the written language represents the spoken language. In alphabetical systems, spellers rely heavily on the phonological rules that designate letter-sound correspondences (Sprenger-Charolles et al., 1998). However, when spelling irregular words, using phoneme-grapheme correspondences does not necessarily yield proper spellings, thus the alphabetic strategy is eventually supplemented by knowledge of the morphological regularities of the orthography. The current study joins recent research in languages from different typologies, suggesting that in learning to spell, children come to exploit grammatical regularities in their language, matching them with large-sized processing units in spelling (Taha and Saiegh-Haddad, 2017; Bar-On and Kuperman, 2019; Breadmore and Deacon, 2019). Taken together, these studies show that both phonological and morphological skills have a reciprocal relationship with spelling development, indicating the need to set aside the classical dual-route approach in favor of an integration of several linguistic dimensions with sensitivity to distributional morpho-orthographic patterns (Casalis, 2018).

Hebrew, like other languages with alphabetic writing systems, does not have a perfect one-to-one phoneme-to-letter relation, nor is it the only language to represent morphology in its orthographic patterns. In fact, orthographies most often ignore phonological differences to express the coarser-grained, semantically grounded generalizations of the morphological system (Berg and Aronoff, 2017). For example, the English adjective suffix *-ic* has three different pronunciations in *electric, electricity*, and *electrician* – *k* in the adjective, *s* in the nominal derived from the adjective preceding the abstract suffix *–ity*, and *š* in the agent noun derived from the adjective preceding the agent suffix *–ian*. All three phonological variations are spelled uniformly by the letter sequence -IC, signifying the adjective suffix. This is not an isolated occurrence: *-ic* adjectives such as *pacific, tactic, basic;* derived- *icity* nominals such as *complicity, felicity, authenticity*, and derived- *ician* agent nouns such as *phonetician, politician, technician* – all reinforcing the consistent relationship between the spelling and pronunciation of the *–ic* suffix in these three morphological classes (Ravid, 2012). Learning the spelling of such morphological families will benefit from the interaction of grammatical meaning, phonological allomorphy, and orthographic consistency (Sandra, 2018). In fact, morphological knowledge has been shown to play a key role in adults’ spelling abilities. Perry et al. (2002) showed that English-speaking adults take into account morphological chunks in assessing “wordlikeness” in spelling judgments: they pointed at morphologically complex non-words as being most wordlike, and did not merely adhere to smaller units guided by phonological considerations. Thus, morphologically motivated orthographic representations can be assumed to exist in the linguistic cognition of mature spellers, and they can serve to facilitate spelling in cases of disrupted phoneme-to-grapheme mapping (Gillis and Ravid, 2006).

The current study traced the developmental route of homophonous affix letter spelling in Hebrew, as reflecting the changing roles of five morpho-orthographic principles – morpho-orthographic transparency, affix letter prevalence, morpho-phonological competition, overtness of the phonological-orthographic link, and phono-morpho-orthographic consistency. Specifically, the present study aimed to investigate whether and when Hebrew-speaking school-going children and adolescents apply these morpho-orthsographic principles in learning to spell, suggesting that they are sensitive to morphology and not just phonology.

Within the affix spelling system, there can be a wide range of structural complexity, from the use of fixed affix sequences or chains to more complex and variable probabilistic patterns that are less predictable. In the current paper we examined children’s grasp of the difference between high and low “morpho-orthographic complexity” of affix letter spelling, as presented in Schiff and Levie (2017). To this end, this study developed a metric to quantify the complexity of different categories of affix spellings, assessing this complexity along two dimensions: (i) word frequency and (ii) affix regularity, transparency and consistency (RTC), as expressed by the five criteria.

The overall picture that emerged from the results is as predicted, indicating a long and protracted learning trajectory of affix letter spelling in Hebrew. Two findings in this study indicate that spelling of affix letters in Hebrew evolves with age: first, the increased accuracy across all affix letters in the spelling task; and second, the changing roles of the five criteria making up the RTC metric. All affixes showed a learning curve that was not over in 7th grade, and in some cases, showed that spelling acquisition of homophonous affix letters was still under way even in high school. These results do not present the same picture as in Ravid (2001); Ravid and Bar-On (2005), and Gillis and Ravid (2006), where homophonous affix letters appeared to be learned in the early years of elementary school. This discrepancy is explained by the two innovations of this study. First, unlike all previous studies on the morphology of Hebrew spelling, this study did not compare root with affix letters, where affix homophones are in general easier to acquire than root homophones; rather, the current study focused on the acquisition of affix letters alone, which allowed us to probe deeper into all factors underlying their learning. And second, the previous studies on homophonous affix letters mainly sampled those with typical behavior – consonantal letters with high morphological prevalence and consistent behavior, in words with demarcated envelopes. These indeed demonstrated very early acquisition in the current study as well. In contrast, the present study examined the full array of Hebrew affix letters, both consonantal and with vowel values, with all of their functions, revealing the differing roles of the five spelling principles in overcoming RTC challenges across the school years.

An important finding in this study is that the higher the complexity and irregularity in spelling, the higher the differences between the lower and higher grade participants. The differentiated reliance on spelling principles across the grade levels in this study demonstrates this effect. We found that 2nd and 4th graders heavily relied on the principle of phono-morpho-orthographic consistency, that is, adhered to the strong generalization of ה marking the end of words with final *a.* This knowledge, already present in kindergarteners (Levin et al., 2001), reflects the high frequency of feminine *a* represented by ה in Hebrew (Ravid and Haimowitz, 2006). However, to achieve correct spelling of all final open syllables, spellers need to note that words of masculine gender ending with *a* violate this generalization, as they are not spelled with a final ה. For example, *katávta* “you, masculine, wrote” is correctly spelled as כתבת, while many 4th graders still spell it erroneously with a final ה as כתבתה. Thus, in 4th grade, young spellers are still challenged by the specific environments where the final ה generalization does not apply. Acquiring this knowledge, at the interface of grammatical gender marking, guttural/pharyngeal phonological segments, and specific orthographic, requires further morphological learning and more experience with written Hebrew.

While already able to overcome the tendency to adhere to morpho-orthographic consistency, 7th graders were still challenged in the current study by two factors – morpho-phonological competition and letter prevalence, as indicated by their spelling patterns. These results reflect ongoing learning of increasingly specific grammatical environments requiring increasingly honed phonological discernment and the ability to relate the autonomous domains of speech and writing (Karmiloff-Smith, 1994; Ravid, 2019b). In the case of morpho-phonological competition, 7th graders need, for example, to spell out the subtle phonological difference between past and future verb prefixes (*h* vs. *y*), which translates to different spellings (ה vs. י), both competing in the same morpho-phonological arena. In the case of letter prevalence, 7th graders need to recruit information about rare spelling/affix matchings, for example final *v* marking plural possessives by ו (*ban-av* “his sons”). While Hebrew-speaking 7th graders have already gained command of a great deal of Hebrew morphology and its written correlates, this study shows that learning of lexically specific, literate, rarer affixes is still under way.

The most challenging affixes in the current study, which did not gain complete mastery even in 10th graders (2 years away from high school graduation) are those in violation of the principles of transparent envelope and overt phonology. These are two extreme cases which fundamentally undermine the phonology-morphology-orthography link that enables the correct spelling of affixes. In the case of non-transparent affix envelopes, the demarcation of the root core from the affix margins is opaque, so that all homophones are treated as having two spelling options. For example, in cases of morphological metathesis such as *histader* “get arranged,” the root *s* exchanges place with the affixal *t*, so that it is not clear whether the *t* is affixal (and thus has only one possible spelling as ת), or a root letter (and thus has two possible spellings as ת or ט). In the case of covert phonology, letters are not linked up with phonological segments, e.g., *-av* spelled with י (normally reflecting the vowel *i*). These extreme violations of affix spelling patterns, which require specialized knowledge of rare morpho-phonological constructions, still challenge 10th graders. This is in line with evidence from non-Semitic studies. For instance, a study conducted among French-speaking first, second and third graders on the acquisition of silent-letter endings (Sénéchal et al., 2016) confirmed that children have difficulty using silent-letter endings when spelling pseudowords, as the absence of phonological cues makes it harder to retrieve the silent forms from memory. The present results suggest that the tendency to use a familiar orthographic form often wins out in spelling production, even in skilled spellers (Treiman et al., 2015).

The results of this study are also in line with previous studies that suggested that frequency is a major factor influencing any inquiry into linguistic skills (Ambridge et al., 2015), including both reading (Weekes et al., 2006) and spelling (Alegria and Mousty, 1996; Lété et al., 2008) development. The study described here indicates that frequency plays a major role in the development of affix spelling as children’s spelling accuracy becomes gradually higher as they progress in age. Our results indicate that in lower grades, frequency is essential to spelling accuracy, but with increased age and spelling experience, performance on non-frequent words improves compared to that of frequent words. Moreover, the difference between the spelling accuracy of words with differing RTC affixation decreased as age and frequency increased. Our interpretation is that older participants have acquired the lexical representations of words with less regular, transparent and consistent affix patterns, and thus were not disadvantaged in spelling these words compared to words with regular affix letters.

The results of this study thus suggest that the typological characteristics of the language and its alphabetic writing system contribute to spellers’ sensitivity to morphology when determining which spelling alternative is correct (Gillis and Ravid, 2006). Similar to our Hebrew-speaking participants, English-speaking and French-speaking children also demonstrated implicit learning of the morphological patterns in their orthography (Treiman et al., 1993; Treiman and Cassar, 1997; Pacton et al., 2001, 2002).

### Theoretical and Applied Implications

One contribution of this study is toward the resolution of the debate regarding the dual/singular model in explaining the results of this spelling study (Holmes and Babauta, 2005). According to our interpretation of the data, the dual-route model may not explain the acquisition of Hebrew affix spelling. While it has been useful in explaining the differences in performance between dichotomous regular and irregular cases in the acquisition of a given linguistic structure, spelling of affix letters in Hebrew goes beyond the regular-irregular dichotomy to include complex features of grammar, phonology, and orthography. The evidence from the present study suggests that children may share a common learning mechanism for spelling complex words. Our analysis demonstrated the impact on spelling performance of all factors - the demarcation of the affix envelope, the prevalence of affix letters in various morphological roles, morpho-phonological competition among morphologically similar affixes, the overtness of the phonological-orthographic link, and the consistency of the phono-morpho-orthographic link. Examining their differential contributions helped us provide a more nuanced account of the development of affix spelling, one that determines to a large extent the sequence and pace at which affix spelling categories are acquired.

The items in our spelling test were all real words, and thus additional work is needed to determine whether morpho-orthographic principles of affix spelling also influence the spelling of non-words. For example, is it more difficult to spell a non-word with a phonologically covert or non-demarcated affix envelope? Another issue for future research concerns the examination of affix spelling acquisition in reading impaired populations, with the ultimate goal of using specific morpho-orthographic principles of affix spelling in diagnosis and remedial instruction.

Although questions remain, our results shed light on the specific characteristics of affix spelling that influence spellers’ choices when more than one option is available. First, Hebrew-speaking children do not acquire accurate spelling of all Hebrew words and structures at the same rate. This goes beyond previous studies, which focused on the difference between homophonous root and affix spelling (Ravid, 2001, 2005): now we know that affixes differ among themselves in the challenges they pose to spellers, and we have been able to capture these differences both theoretically and empirically. Secondly, we have shown that spellers in this study became less sensitive to frequency distributions as they become older. This indicates that Hebrew affix spelling is indeed morpho-lexical in nature: with age and schooling, older children and adolescents expand their mental lexicons to include less frequent items, more abstract and lexically specific words, and more morphologically complex words with more and different affixes. Thirdly, we have seen that our participants relied on different phonological, morphological and orthographic knowledge at different stages of their affix spelling development. This means that the consolidation of a qualitative knowledge base of affix spelling is part of the period of Later Language Development (the school years), and is tightly linked to the development of mature and coherent links between phonology, lexicon, and grammar. Finally, acquisition of correct affix spelling of a word clearly depended on the complexity of the spelling pattern being acquired in terms of the metrics that we first introduced in this novel study.

Taken together, the accumulated evidence suggests that even though phonology is a major factor in spelling acquisition (Bosman and Van Orden, 1997), and sound-to-symbol mapping represents a vital self-instruction process for processing new words (Ehri, 1992; Share, 1999), young children also have to rely on morphological knowledge, if they are to select between different spellings (Angelelli et al., 2014; Treiman, 2017) of a word. These characteristics of affix spelling may not necessarily be exclusive to Hebrew and could be relevant to a wide array of languages and their orthographies.

Moreover, applied implications can be gained from this study for our understanding of how Hebrew morphology is learned and in particular, how the developmental path of learning to spell involves complex morphological structures. As the Hebrew lexicon and grammar are strongly organized by morphological principles (Schiff and Raveh, 2007; Ravid, 2012; Schiff et al., 2016; Ashkenazi et al., 2019), these findings have clear clinical and educational ramifications. First and foremost, these results strongly suggest that rigorous morphological instruction is necessary in teaching children and adolescents to identify and use morphological cues in spoken and written Hebrew. Even more importantly, we now have a solid knowledge base regarding the acquisition route of all classes of affixes classified in phonological, morphological and lexical categories. This body of knowledge can be used to inform teachers of emphases in their spelling instruction and to enable clinicians to focus on specific categories in response to children’s persistent error patterns. In sum, the well-motivated, detailed, empirically endorsed information this study provides can thus be of immense value to educators, remedial teachers, educational psychologists, and speech-language pathologists.

## Data Availability Statement

The datasets generated for this study are available on request to the corresponding author.

## Ethics Statement

The studies involving human participants were reviewed and approved by the Ministry of Education (Israel) and the Bar Ilan Ethics Committee. Written informed consent to participate in this study was provided by the participants’ legal guardian/next of kin.

## Author Contributions

SR is an MA student whose Master’s thesis was jointly directed by RS and DR. Data was collected by SR. RS and DR wrote the manuscript together.

## Conflict of Interest

The authors declare that the research was conducted in the absence of any commercial or financial relationships that could be construed as a potential conflict of interest.
